# Communication challenges experienced by migrants with cancer: A comparison of migrant and English‐speaking Australian‐born cancer patients

**DOI:** 10.1111/hex.12529

**Published:** 2017-03-05

**Authors:** Amelia Hyatt, Ruby Lipson‐Smith, Penelope Schofield, Karla Gough, Ming Sze, Lynley Aldridge, David Goldstein, Michael Jefford, Melanie L. Bell, Phyllis Butow

**Affiliations:** ^1^ Department of Cancer Experiences Research Peter MacCallum Cancer Centre Melbourne Vic. Australia; ^2^ Department of Psychology School of Health Sciences Faculty of Health, Arts and Design Swinburne Melbourne Vic. Australia; ^3^ Sir Peter MacCallum Department of Oncology The University of Melbourne Parkville Vic. Australia; ^4^ Melbourne School of Psychological Sciences Faculty of Medicine, Dentistry, and Health Sciences The University of Melbourne Parkville Vic. Australia; ^5^ Department of Nursing Faculty of Medicine, Dentistry, and Health Sciences University of Melbourne Parkville Vic. Australia; ^6^ School of Psychology University of Sydney Sydney NSW Australia; ^7^ Psycho‐Oncology Cooperative Research Group University of Sydney Sydney NSW Australia; ^8^ Prince of Wales Hospital Sydney NSW Australia; ^9^ Prince of Wales Clinical School University of New South Wales Sydney NSW Australia; ^10^ Division of Epidemiology and Biostatistics Mel and Enid Zuckerman College of Public Health University of Arizona Tucson AZ USA; ^11^ Centre for Medical Psychology and Evidence‐Based Decision‐Making School of Psychology University of Sydney Sydney NSW Australia

**Keywords:** cancer, communication, health literacy, healthcare, migrant, oncology

## Abstract

**Objectives:**

Understanding the difficulties faced by different migrant groups is vital to address disparities and inform targeted health‐care service delivery. Migrant oncology patients experience increased morbidity, mortality and psychological distress, with this tentatively linked to language and communication difficulties. The objective of this exploratory study was to investigate the communication barriers and challenges experienced by Arabic, Greek and Chinese (Mandarin and Cantonese) speaking oncology patients in Australia.

**Methods:**

This study employed a cross‐sectional design using patient‐reported outcome survey data from migrant and English‐speaking Australian‐born patients with cancer. Patients were recruited through oncology clinics and Australian state cancer registries. Data were collected regarding patient clinical and demographic characteristics and health‐care and communication experiences. Data from the clinics and registries were combined for analysis.

**Results:**

Significant differences were found between migrant groups in demographic characteristics, communication and health‐care experiences, and information and care preferences. Chinese patients cited problems with understanding medical information, the Australian health‐care system, and communicating with their health‐care team. Conversely, Arabic‐ and Greek‐speaking patients reported higher understanding of the health‐care system, and less communication difficulties.

**Conclusions:**

Our study findings suggest that migrant groups differ from each other in their health communication expectations and requirements. Lower education and health literacy of some groups may play a role in poorer health outcomes. Public health interventions and assistance provided to migrants should be tailored to the specific needs and characteristics of that language or cultural group. Future research directions are discussed.

## 1 INTRODUCTION

In 2015, the number of migrants worldwide reached 244 million,^1^ and by 2015, global displacement reached levels higher than post‐World War II.^2^ Most nations now face the challenging task of providing appropriate and equitable health care to migrant and asylum‐seeking populations.

Disparities in morbidity and mortality outcomes for migrants with non‐communicable diseases such as cancer compared with local populations are widely documented.^3^, ^4^, ^5^, ^6^, ^7^, ^8^ Survival in migrant populations is still worse after controlling for socio‐demographic factors such as income, poverty level and education;^6^, ^8^ anxiety and depression is higher, and health‐related quality of life is lower.^6^, ^8^, ^9^, ^10^ Additionally, high levels of distress in migrant populations are not found when the same populations are studied in their home countries, suggesting that certain characteristics of being a migrant could be responsible.^9^

Self‐reported challenges of migrants within their new health‐care setting include the following: difficulties with language, and understanding or navigating of local health‐care systems,^10^, ^11^, ^12^, ^13^, ^14^, ^15^ experiences of discrimination, lack of cultural sensitivity and understanding from medical professionals 10, 11, 16, 17 and disappointment due to expected vs perceived care.^12^ There are indications that such experiential challenges are linked to higher levels of psychological distress in migrant patients with cancer.^13^, ^18^, ^19^ Other factors that may contribute to disparities in health outcomes include inaccurate understanding of their diagnosis,^20^ and misunderstandings regarding the causes of cancer,^15^, ^16^, ^17^ which have both been shown to be comparatively higher in migrant patients.

The term migrant covers individuals who may hail from a multitude of different countries and cultures. Migrant groups are likely to differ from each other on many dimensions, such as education and literacy, income and access to insurance, exposure to trauma and time spent in the new home country. It is well established through epidemiological research that there are differences in cancer morbidity and mortality between migrant language and cultural or location groupings due to environmental and behavioural influences on the aetiology of various cancers.^21^, ^22^, ^23^, ^24^, ^25^ Accordingly, psychosocial and experiential similarities and differences need to be taken into account when determining which factors underlie disparities in health‐care outcomes. Awareness of the similarities and differences between migrants groups will allow for development of tailored interventions and targeted assistance. Indeed, our Australia‐based research group has previously found that Arabic and Greek migrants with cancer report higher levels of anxiety compared to their Chinese counterparts, who report levels of anxiety equivalent to English‐speaking Australian‐born patients,^26^ indicating that a greater proportion of Arabic‐ and Greek‐speaking migrants than Chinese‐speaking migrants would benefit from interventions to reduce anxiety.

Understanding the difficulties faced by different migrant groups is therefore vital to inform targeted, evidence‐based policies and service delivery in health care.^27^ The aim of this study was to explore the communication challenges faced by different migrant groups diagnosed with cancer in Australia to determine areas to target for intervention and improvement. We aimed to investigate clinical and demographic characteristics, experiences of communication within the health‐care setting and understanding of the health‐care system for three large migrant groups and an English‐speaking Australian‐born group for comparison.

## 2 METHOD

This study utilized data from two previous studies conducted as part of a research programme investigating patient‐reported outcomes and experiences of migrant and English‐speaking Australian‐born patients with cancer. For the first study, potential respondents were identified via the Australian state Cancer Registries in New South Wales, Queensland and Victoria.^28^ For the second study, potential respondents were identified though 16 oncology clinics in the same three Australian states.^29^ Data from the two studies were deliberately combined to enhance the generalisability of our results to the population of interest, reflecting the communication experiences of migrants across the cancer trajectory, from treatment commencement to survivorship. Both studies utilized a cross‐sectional survey design. Consumer advisors for each language group reviewed both study procedures and materials, and provided advice regarding recruitment strategies.^28^, ^29^ The methods relevant to each study are outlined in brief below and detailed in full in the publications referenced above. These studies were approved by all relevant ethics committees.

### 2.1 Participants

A total of 1441 patients were recruited: 596 patients via the state cancer registries and 845 patients via oncology clinics with a response rate of 26% for patients recruited via cancer registries and 62% for patients recruited via oncology clinics.^28^, ^29^ Patients were eligible to participate if they were English‐speaking Australian‐born or if they were born in a country where Arabic, Greek or Chinese is spoken, and spoke that language. Chinese‐, Arabic‐ and Greek‐speaking patients were selected because they are prevalent migrant groups in Australia.^30^ Potentially eligible participants were identified by cancer registry or clinic lists using surnames as an indicator of ethnicity. As per onomastics procedure, first and last name searches were made, and then ethnicity (country of birth and parent/grandparent country of birth) and language/s spoken were confirmed directly with patients. Other eligibility criteria for registry‐identified patients included the following: diagnosis of 1 of the 12 most common cancers (all stages) 1‐6 years previously; aged 18‐80 years at the time of diagnosis; and having a treating doctor assigned to their registry record. Other eligibility criteria for clinic‐identified patients included diagnosis with 1 of the 12 most common cancers (all stages) 0‐12 months previously, aged 18‐80 years at time of recruitment and had commenced treatment at least 4 weeks earlier. In the remainder of this paper, participants are referred to as belonging to “language groups” rather than migrant groups as some analyses include Australian‐born English‐speaking patients.

### 2.2 Procedure

Arabic, Greek and Chinese community advisory groups reviewed study procedures and materials, and provided advice regarding recruitment strategies and interpretation of results. A total of 2307 patients were identified as eligible in the registry study, and 693 of these patients consented, via telephone, to be mailed the survey. Out of a total 1603 potentially eligible patients identified from clinic lists, 1603 patients were approached by staff in outpatient clinics and a total of 1603 patients consented to be mailed a survey. Upon consent, participants in both studies were posted a questionnaire in their primary language. Non‐returnees were telephoned up to three times at different times of the day before being listed as non‐respondents.

### 2.3 Measures

All measures were translated into Arabic, Greek and Chinese using the European Organisation for Research and Treatment of Cancer Translation Protocol.^31^

#### 2.3.1 Clinical and demographic characteristic questionnaire

Patient‐reported demographic and clinical details were collected including age at participation, age at diagnosis, date of diagnosis, primary cancer type, stage of disease at diagnosis, years lived in Australia, marital status, employment status and education level. For the cancer registry study, demographic and clinical details were obtained directly from the registry, including age at diagnosis, date of diagnosis, primary cancer type, disease extent and diagnosis, and measures of rurality and socio‐economic status based on postcode at diagnosis. A measure of socio‐economic status was calculated using the Index of Relative Socio‐economic Advantage and Disadvantage (IRSAD), which indicates relatively greater disadvantage (low scores) or relative lack of disadvantage (high scores) based on the economic and social conditions of people within a geographical area.^32^

#### 2.3.2 Patient‐reported health‐care experiences and preferences scale

Patients’ experiences of communication within the health‐care system and their understanding of the health‐care system were assessed with custom measures. Purpose‐built measures were used as there were no existing scales measuring immigrant‐specific communication variables. The items were developed, piloted on a small sample, and then implemented in the current studies.

Participants rated their understanding of the Australian health system and their understanding of aspects of their illness and care on four‐point and five‐point Likert scales (detailed in Table 2). Some items were provided to migrant participants only. These included confidence speaking English and communicating with hospital staff, understanding of the health‐care system, interaction with and preferences for interpreters, perception of health‐care quality, and decision‐making preferences. Questions regarding confidence speaking and understanding English were worded slightly differently for registry and hospital participants, as the studies were not conducted concurrently. For registry participants, the question referred to confidence speaking with all hospital staff. For hospital participants, one question addressed confidence speaking to doctors, while another addressed confidence speaking to hospital staff other than doctors and nurses. A full listing of items and their response scales appears in Tables 2 and 3.

### 2.4 Statistical analysis

Descriptive statistics were used to summarize demographic and clinical characteristics by language group. Analysis of variance was used to compare all language groups on age at participation, age at diagnosis, years lived in Australia and IRSAD scores. Results for both registry and clinic participants were analysed together, except where the wording of the response options differed between the samples. Chi‐squared tests were used to compare all language groups on their understanding of the health‐care system and to compare migrant language groups on their communication difficulties, confidence with English, experience using interpreters and perception of care. For significant chi‐squared tests, adjusted standardized residuals were used to identify cells with smaller or larger counts than would be expected, had there been no association between tested variables.

All statistical analyses were conducted using SAS. Alpha was set at 0.05 (two‐tailed) for all relevant tests. No adjustment was made for multiple testing, as this was an exploratory study with no pre‐specified hypotheses.^33^

## 3 RESULTS

### 3.1 Participants

Demographic and clinical characteristics by language group are provided in Table 1.

**Table 1:** Demographic and clinical characteristics by language group

Characteristics, *n* (%)	English‐speaking Aust.‐born*n*_Reg_=319*n*_Clinic_=274	Arabic‐speaking migrant*n*_Reg_=57*n*_Clinic_=145	Chinese‐speaking migrant*n*_Reg_=141*n*_Clinic_=248	Greek‐speaking migrant*n*_Reg_=79*n*_Clinic_=178	*P*‐value^a^
Gender					
Male	271 (46)	90 (45)	164 (42)	143 (56)	.007
Female	322 (54)	112 (55)	225 (58)	114 (44)	
Marital status					
Married or partnered	422 (71)	149 (74)	307 (79)	198 (77)	.04
Not married or partnered	171 (29)	53 (26)	82 (21)	59 (23)	
Education					
Did not complete high school	37 (6)	43 (22)	49 (13)	137 (54)	<.001
High school/tech college	443 (73)	112 (56)	196 (50)	99 (39)	
University	111 (19)	45 (23)	144 (37)	16 (6)	
Cancer type					
Breast	185 (31)	76 (38)	130 (33)	63 (25)	.004
Colorectal	108 (18)	34 (17)	60 (15)	37 (14)	
Prostate	97 (16)	27 (13)	39 (10)	38 (15)	
Leukaemia, lymphomas	51 (9)	15 (7)	38 (10)	34 (13)	
Lung	48 (8)	17 (8)	54 (14)	26 (10)	
Bladder, kidney	27 (5)	3 (1)	5 (1)	7 (1)	
Head and neck	7 (1)	2 (1)	11 (3)	5 (2)	
Other	70 (12)	28 (14)	52 (14)	47 (18)	
Disease stage					
Early	402 (68)	132 (66)	245 (64)	160 (65)	
Late	95 (16)	36 (18)	82 (21)	56 (23)	
Unknown/not applicable/missing	91 (15)	31 (16)	55 (14)	32 (13)	
Currently on treatment					
Registry sample	17 (5)	7 (12)	9 (6)	7 (9)	
Clinic sample	201 (74)	100 (70)	157 (64)	123 (72)	
Age^a^, mean (SD)	63 (11.3)	61 (11.8)	59 (12.6)	68 (9.5)	<.001
Age at diagnosis^a^, mean (SD)	60 (11.4)	58 (12.7)	56 (13.7)	65 (10)	<.001
Years in Australia^a^, mean (SD)	63 (11.6)	27 (15.5)	20 (11.1)	46 (8.6)	<.001
IRSAD, mean (SD)	1019 (76)	991 (71)	1050 (79)	1024 (71)	<.001

*n*_Reg_, number of participants in the registry sample; *n*_Clinic_, number of participants in the clinic sample; IRSAD, Index of Relative Socio‐economic Advantage and Disadvantage.

a Analysis of variance comparison between all groups.

There were no statistically significant differences between language groups in disease stage, whether currently on treatment, or months since diagnosis. However, there were significant differences between language groups in cancer type: specifically, fewer Greek‐speaking participants had a diagnosis of breast cancer, more Chinese‐speaking patients were diagnosed with lung cancer and fewer with prostate cancer, and English‐speaking Australian‐born patients had more than expected diagnosis of prostate, bladder, kidney, and lung cancers.

Significantly more Greek‐speaking respondents were male (56% vs 42%‐46% in the other language groups). More Chinese‐speaking and fewer English‐speaking Australian‐born respondents were married or partnered (79% vs 71%). Chinese‐speaking respondents were the most highly educated group, with 37% indicating that they had completed a university degree. In contrast, English‐speaking Australian‐born respondents were more likely to indicate that they had completed high school or technical college (73%) and Greek‐speaking respondents that they had not completed high school (54%). Approximately half of the Arabic‐speaking respondents had completed high school or technical college (56%); however, there was a fairly even split between Arabic‐speaking patients who had not completed high school education and who had completed university, 22% and 23% respectively.

Age, IRSAD, age at diagnosis, and years lived in Australia also differed significantly between language groups. Chinese‐speaking respondents were significantly younger and Greek‐speaking respondents were significantly older than other groups, both in general and at age of diagnosis. Arabic‐speaking patients scored significantly lower on the IRSAD, indicating relatively greater socio‐economic disadvantage, whereas Chinese‐speaking patients scored significantly higher, indicating relatively greater socio‐economic advantage. Greek‐speaking respondents had lived in Australia the longest of all the migrant groups and Chinese‐speaking the shortest.

### 3.2 Health‐care understanding, perceptions and experiences

The relationship between language groups and health‐care understanding, perceptions, and experiences are provided in Table 2. Responses to most variables were significantly different between language groups; mostly due to differences between English‐speaking Australian‐born and Chinese‐speaking respondents.

**Table 2:** Understanding, perceptions, and experiences of health‐care by language group

Variables, *n* (%)	English‐speaking Aust.‐born*n*_Reg_=319 *n*_Clinic_=274	Arabic‐speaking migrant*n*_Reg_=57*n*_Clinic_=145	Chinese‐speaking migrant*n*_Reg_=141*n*_Clinic_=248	Greek‐speaking migrant*n*_Reg_=79*n*_Clinic_=178	*P*‐value^a^
Understanding of the health‐care system^b^	529 (90)	163 (81)	211 (55)	180 (70)	<.0001
Understand the reasons for my tests^b^	560 (95)	179 (90)	312 (81)	227 (90)	<.0001
Understand the effects of treatment on my body^b^	531 (90)	179 (89)	316 (81)	214 (87)	.0009
Understand the effects of treatment on my life^b^	491 (83)	162 (81)	316 (83)	193 (79)	.5
Understand the effects of treatment on my family life^b^	480 (82)	155 (78)	312 (82)	193 (79)	.5
Amount of information patient felt was given					
Too much	29 (5)	26 (13)	19 (5)	55 (22)	<.0001
Right amount	524 (88)	163 (81)	312 (81)	171 (68)	
Too little	40 (7)	12 (6)	55 (14)	24 (10)	
Patient discussed the advantages and disadvantages of treatment with doctor^c^	516 (89)	169 (88)	226 (63)	213 (87)	<.0001
Decision‐making preferences^d^					
Mostly doctor's decision	279 (47)	114 (58)	269 (70)	130 (52)	<.0001
Equal patient and doctor decision	307 (52)	77 (39)	111 (29)	113 (45)	
Mostly patient's decision	7 (1)	7 (4)	7 (4)	7 (3)	
Treatment decision involvement with doctor					
Patient felt more involved than they wanted	11 (2)	21 (11)	47 (13)	130 (52)	<.0001
Patient felt involved as they wanted	556 (94)	165 (83)	274 (74)	113 (45)	
Patient felt not as involved as they wanted	25 (4)	13 (7)	50 (13)	7 (3)	
Family's involvement in treatment decision‐making^e^					
Family mostly involved in decision	15 (3)	15 (8)	27 (7)	16 (6)	<.0001
Patient and family were equally involved	465 (80)	160 (82)	300 (81)	211 (85)	
Patient only involved	101 (17)	20 (10)	42 (11)	20 (8)	

*n*_Reg_, number of participants in the registry sample; *n*_Clinic_, number of participants in the clinic sample.

a Chi‐squared comparison between groups.

b Responded “well” or “very well” on a four‐point Likert scale. Other responses were “not so well” and “not well at all.”

c Responded “yes” to a choice of “yes”, “no”, or “not sure/can't remember.”

d Responded to a five‐point Likert scale with the following options: “the doctor should make the decision using all that is known about the treatment” or “the doctor should make the decisions but strongly consider my needs and priorities”, “the doctor and I should make the decisions together on an equal basis”, “I should make the decisions, but strongly consider the doctors opinion”, or “I should make the decision using all I know or learn about the treatments”.

Responded to a five‐point Likert scale with the following options: “the doctor should make the decisions using all that is known about the treatments”, “the doctor should make the decisions but strongly consider my needs and priorities”, “the doctor and I should make the decisions together on an equal basis”, “I should make the decisions, but strongly consider the doctor's opinion”, or “I should make the decision using all I know and learn about the treatments”.

e Responded to a six‐point Likert scale giving the following options: “it is my family's role to make the decision for me”, “I want to tell my family my opinions and feelings regarding my options and let them decide”, “I want to come to a decision together with my family”, “I want to make the decision after I have heard the opinions of my family”, “I want to make the decision without involving my family”, or “Not relevant, have no family to help me decide”.

Significantly fewer Chinese‐speaking respondents and more English‐speaking Australian‐born respondents reported understanding the health‐care system, reasons for tests, and possible treatment effects on their body (see Table 2). Additionally, significantly fewer Chinese‐speaking respondents had discussed the advantages and disadvantages of treatment with their doctor (63% vs 87%‐89% in the other language groups).

More English‐speaking Australian‐born respondents and fewer Greek‐speaking respondents felt they had been given the right amount of verbal or written information (88% and 68% respectively). More Greek‐speaking respondents felt that they had been given *too much* information (22%) than in the other language groups (5%‐13%). Conversely, more Chinese‐speaking respondents felt that they had been given insufficient information (14%).

### 3.3 Confidence with English, professional interpreter services and perception of health‐care quality

Responses to items assessing confidence with English, professional interpreter services, and perceptions of quality of care by language group are provided in Table 3.

**Table 3:** Confidence with English, professional interpreter services, and quality of care by migrant language group

Variable, *n (%)*	Total *n* a	Arabic‐speaking migrant *n*_Reg_=57*n*_Clinic_=145	Chinese‐speaking migrant*n*_Reg_=141*n*_Clinic_=248	Greek‐speaking migrant*n*_Reg_=79*n*_Clinic_=178	*P*‐value^b^
How confident the patients felt with English					
Patient confident speaking English with hospital staff^c^	276	45 (79)	94 (67)	56 (72)	.2
Confident understanding English spoken by hospital staff^c^^,^ e	276	44 (77)	90 (64)	54 (69)	.2
Confident speaking English with hospital staff other than doctors and nurses^d^^,^ e	567	90 (63)	111 (45)	114 (64)	<.0001
Confident understanding English spoken by hospital staff other than doctors and nurses^d^^,^ e	567	93 (65)	107 (44)	113 (63)	<.0001
Patient had difficulty communicating with doctors in English	839	127 (63)	287 (75)	155 (61)	.0002
Patients identified needing someone (family or professional interpreter) to interpret at medical visits	843	106 (53)	219 (56)	112 (44)	.0006
When using Professional Interpretation^g^ patients found that they were					
Confident with accuracy of the interpreter	320	65 (92)	176 (92)	51 (89)	.9
Comfortable with interpreter	321	64 (91)	173 (90)	49 (85)	.4
The interpreter explained medical terminology well	319	62 (87)	163 (86)	54 (93)	.3
Whether patients were able to access the same interpreter every time					
No, but were not bothered by this	311	39 (57)	135 (72)	36 (65)	.1
No, but were bothered by this		13 (19)	29 (16)	8 (15)	
Yes, same interpreter was used each time		17 (25)	23 (12)	11 (20)	
Patient perceived quality of care^f^					
Care was the same or better because of cultural background	838	198 (99)	375 (97)	250 (99)	.02
Care was worse because of lack of English	635	12 (9)	44 (15)	14 (7)	.03

*n*_Reg_, number of participants in the registry sample; *n*_Clinic_, number of participants in the clinic sample.

a *n* varies due to separate reporting of hospital and registry studies when items were worded differently or due to conditional responses (e.g. “only answer if you had an interpreter present at the appointment”).

b Chi‐squared comparison between groups.

c Registry patients only.

d Clinic patients only.

e Responded to a four‐point Likert scale with the following options: “Very confident”, “confident”, “not so confident”, “not confident at all.”

f Responded to a four‐point Likert scale with the following options: “Not at all”, “sometimes”, “often”, “very often.”

g Including only patients who used an interpreter.

Significantly more Chinese‐speaking respondents reported difficulties communicating with doctors in English (75% vs 61% and 63% for Greek‐ and Arabic‐speaking respondents respectively). Additionally, when reviewing responses from oncology clinic participants alone, associations between language group and confidence speaking and understanding English were significant. Fewer Chinese‐speaking respondents were confident speaking (45%) and understanding (44%) English when communicating with hospital staff other than doctors or nurses compared with Arabic‐ and Greek‐speaking respondents. Conversely, associations between language group and confidence in English were not significant for registry participants, possibly due to the wording of the question for this sample.

More Chinese‐speaking and fewer Greek‐speaking respondents reported that they needed a professional to interpret at medical visits “sometimes” or “often” (56% vs 44%); however, the wording of the question may have excluded some patients who used a family member for interpretation. Language group was not associated with perceptions of interpretation quality. The majority of respondents felt that they were confident with the accuracy of their interpreter (89‐92%) and explanations of medical terminologies (86‐93%) and felt comfortable with the interpreter (85%‐91%).

Among respondents who self‐assessed as not speaking English well, the majority felt that they had received “the same” or “better” care regardless of their cultural background (97%‐99%). However, significantly more Chinese‐speaking respondents believed that the quality of care they received was worse because of their lack of English (15% vs 9% and 7% for Arabic‐ and Greek‐speaking respondents respectively).

## 4 DISCUSSION

This study aimed to explore and identify experiences of communication within the health‐care system of migrant groups diagnosed with cancer in Australia, by looking at some of the most prevalent language groups in the country. Our findings demonstrated that Arabic‐, Greek‐ and Chinese‐speaking patients differ from each other in terms of demographic and clinical characteristics, their experience of communication within the health‐care setting and their understanding of the health‐care system. Evidence of these differences allows for identification and implementation of more targeted health‐care improvements and outcomes for migrant groups in the oncology setting.

### 4.1 Arabic, Greek and Chinese migrants in Australia

The findings from our exploratory study suggest what may be the typical clinical and demographic characteristics of certain migrant cancer patient groups in Australia. Our data were combined from two different studies employing two different sampling methods, so our sample is likely to be a fair representation of the Arabic, Greek and Chinese migrant populations with cancer in Australia. Significant group differences in age, sex, education, and socio‐economic status arguably reflect different situations, times, and reasons for migration to Australia. Many people migrated from Greece to Australia after World War II. Greek migrants in the sample were predominantly male and less likely to have completed formal education, with just over half not completing high school. Greek patients also were older at age of diagnosis, and as a cohort in general. Many Greek migrants had lived in Australia for the majority of their lives, almost double the average years in Australia of the Arabic and Chinese cohorts. The Civil War in Lebanon from 1975 to 1990s led to the migration of many Arabic‐speaking people, and migration has continued from many Arabic‐speaking nations leading to great diversity in the Arabic‐speaking community in Australia.^34^ The Arabic patient sample, like the Chinese, had slightly more females than expected, were younger in age and at diagnosis, and had lived in Australia almost half the time of the Greek‐ and English‐speaking Australian‐born populations. However, large standard deviations indicate large variance within the Arabic‐speaking sample, particularly regarding years lived in Australia. Arabic patients also had the lowest score on the IRSAD. The end of the “White Australia policy” in 1965 (which permitted only European migrants to Australia), and asylum offered to Chinese students studying in Australia to settle permanently from the late 1980s due in part to the massacre in Tiananmen Square, encouraged the migration of Chinese‐speaking people to Australia in the later part of last century.^34^ Indeed, Chinese‐speaking participants reported higher educational levels and socio‐economic advantage compared to the other migrant groups and have lived in Australia for less time on average, which may be a reflection of the tertiary education‐driven migration from China.

Understanding of demographic trends within specific migrant groups, and differences between groups, can have implications for health promotion and prevention strategies and interventions to improve health‐care delivery and health outcomes. Some examples are as follows: significant differences in sex prevalence within a migrant group could inform increased information and awareness around screening for male/female‐specific cancers; knowledge of lower education in certain groups could lead to targeted health literacy interventions in specific migrant populations; and knowledge of lower socio‐economic status may flag other risk factors for particular migrant groups. Additionally, identification of demographic trends in particular migrant groups can highlight the cultural differences between groups that should be considered in health‐care delivery, for example: the relationship between ethnicity, smoking, and oral cancer;^35^, ^36^ cultural‐specific food and higher cancer incidence;^37^ or exposure to communicable diseases such as hepatitis C and cancer risk.^38^ These demographic differences between migrant groups highlight the pitfalls of regarding all migrants as a homogenous group. Identification and understanding of trends identified within different migrant groups can assist with the development and delivery of more targeted health‐care approaches, providing better cost‐efficiency and outcomes.

### 4.2 Communication difficulties and differing health‐care paradigms

These results are consistent with the results of previous literature which found that limited English proficiency was related to lower levels of medical comprehension and reduced communication of important medical information.^13^, ^18^, ^19^ In our study, we found that the majority of participants in all migrant groups reported difficulty communicating with the health‐care team in English. However, when comparing between migrant groups, Chinese patients in particular cited more problems with understanding the reasons for tests, the physical impact of treatment, and the Australian health‐care system in general. Confidence speaking with hospital staff also varied between language groups, with Chinese‐speaking migrants significantly more likely to report less confidence than other language groups regarding speaking with and understanding doctors, nurses and other hospital staff.

The disparity between English‐speaking Australian‐born and Chinese‐speaking patients in both their understanding of the health‐care system and confidence in communication is consistent with previous literature.^19^, ^39^ Interestingly, Chinese patients were more likely to be high school or tertiary educated, suggesting that understanding of the health‐care system is not necessarily reflective of level of education.^40^

The traditional Chinese medical paradigm is notably different to that of Western medicine.^41^, ^42^, ^43^ Many Chinese‐speaking migrants in Australia must adjust to a new and different way of thinking about health care, interaction with their health‐care team and the relationship between their body, their illness, and the treatment they receive.^16^, ^42^, ^43^, ^44^, ^45^ Chinese‐speaking migrants may therefore face a cultural shift in health‐care, which may explain their particular difficulty in understanding the Australian health‐care system. In addition, Chinese‐speaking migrants in this study had lived less time in Australia than the Arabic‐ and Greek‐speaking migrants and so may have been less acculturated and less familiar with the Australian health‐care system than the other migrant groups.

Importantly, the Arabic‐ and Greek‐speaking patients in our study reported quite a high understanding of the health‐care system (although still lower than their English‐speaking Australian‐born counterparts), which contrasts with our findings within the Chinese‐speaking patients mentioned above, as well as with the literature that reports low understanding of the health‐care system in migrants in general. Variation in reported understanding of the health‐care system may be a reflection of time lived in Australia (and consequent exposure to the Australian health‐care system). However, this explanation does not account for Arabic‐speaking patients as there is a wide variation in length of time spent in Australia in this cohort. Encouragingly, the majority of patients felt that their care was not affected by a lack of English skills, except a small but noteworthy minority of Chinese‐speaking patients. Further investigation would be beneficial to better understand and provide more appropriate assistance for these patients.

### 4.3 Limited education may be an important predictor of communication difficulties

Greek‐speaking patients, who as a cohort had significantly lower reported education levels, were more likely to report being given too much information, while Chinese‐speaking migrants, whose cohort reported significantly higher education levels, were more likely to report being given too little information. Conversely, other research has found that for English‐speaking patients, low education is linked to a preference for more information.^46^ Medical terminology can be challenging even for those who speak English fluently,^15^ and low education may be linked to poor health literacy.^14^ Further research is needed to determine whether the varied experiences between migrant groups of receiving more or less medical information is a product of differences in education levels, language difficulties, or some other moderating variable such as health literacy.

### 4.4 Communication difficulties may not always result in anxiety

Previous research has indicated that communication difficulties may result in higher levels of anxiety and psychological distress in migrants.^3^, ^16^, ^28^, ^47^ However, this relationship may be more complex than first thought. As discussed above, when exploring communication experiences of migrant groups, Chinese‐speaking patients reported significantly greater communication difficulties than Greek‐ or Arabic‐speaking patients. From confidence with speaking and understanding English, to communicating with their health‐care team, Chinese‐speaking patients reported greater communication problems and challenges. Previous assessment of anxiety in the same cohort within the Australian oncology context discovered that Chinese‐speaking patients reported the same, or lower anxiety than English‐speaking Australian‐born patients,^26^ whereas Greek‐ and Arabic‐speaking patients reported much higher levels of anxiety. Anxiety in migrants with cancer may therefore be due to a complex combination of factors, and not just communication difficulties. Further research would be useful to determine how these factors interplay to produce high or low anxiety in migrant patients with cancer, and to provide recommendations for future assistance for these groups.

### 4.5 Strengths and limitations

Strengths of this study include a large sample recruited from a broad variety of sources. The study assessed migrants as separate groups according to both ethnic background and language spoken. Consumer reference groups for each migrant group reviewed study design and processes for cultural appropriateness.

Last name identification for identifying non‐English‐speaking patients is a potential limitation of the study due to possible inaccuracies. However, patients were asked to verify ethnic background and language spoken, and were excluded if they did not confirm their language group. Another potential limitation is that patients were classified by language rather than by country of origin. This is particularly pertinent for the Arabic‐speaking cohort of patients, which represents a combination of migrants from many different countries and cultures. Further, the many Chinese dialects, the most prominent being Mandarin and Cantonese, were classified together. There were also minor differences in the wording of some items between the registry and outpatient samples. Different outcomes between the language groups may have been due to this methodological anomaly rather than experiential or cultural differences in communication and health‐care knowledge. Measures also did not undergo psychometric testing. Finally, no adjustment was made for multiple testing, as this is not strictly required in exploratory studies. Results should therefore be interpreted cautiously until examined in a confirmatory study.

## 5 CONCLUSION

The results of this study suggest that migrant language groups differ in terms of the issues they face and their health communication requirements. Future research should attempt to address and understand the differences between and within migrant groups in order to assist in the optimal allocation of resources to create targeted health‐care education, screening and health service delivery for people with cancer.

## CONFLICT OF INTERESTS

No conflict of interest relevant to the study. David Goldstein: Bayer, Glaxo, Pfizer (C/A); Celgene (RF, SAB). The other authors indicated no financial relationships.
